# Electrical properties and surface roughness of carbon fibre reinforced polymer under progressive surface abrasion

**DOI:** 10.1016/j.dib.2026.112915

**Published:** 2026-06-01

**Authors:** Muhammad Osama, Catherine E Jones, Bruce Stephen

**Affiliations:** Institute for Energy and Environment, Department of Electronic and Electrical Engineering, University of Strathclyde, 204 George Street, Glasgow G1 1XW, UK

**Keywords:** Carbon fibre reinforced polymer, Electrical resistance, Controlled destructive testing

## Abstract

Two key technologies to reduce aircraft emissions are lightweight structures and the electrification of propulsion systems. Achieving high power density is a barrier to aviation electrification. Carbon fibre reinforced polymer (CFRP) is used in over 50% of state-of-the-art aircraft structures due to its lower density. CFRP offers ten times higher strength depending on the fibre and superior mechanical properties compared to aluminium. Replacement of metallic equipment casings and cable raceways with CFRP offers the opportunity to increase power density. However, the use of CFRP for these applications will give rise to new failure modes where the CFRP forms part of the electrical bonding network (pathway to current return) on the aircraft. Historically, the most common failure mode for aircraft systems is a short circuit due to abrasion of cable insulation. For electrical failures through CFRP, the outer layer of epoxy must also be abraded for an electrical connection to be made between the metallic cable conductor and the carbon fibres. The electrical resistance added to the fault path by CFRP components will vary with the level of abrasion on the surface of the CFRP. This paper describes the capture of datasets [1] which correlate the change in electrical resistance for unidirectional [0°] and woven CFRP with the level of surface abrasion. This data enables users to characterise the failure mode regarding impact on electrical fault response and subsequent resilient electrical power system design and may facilitate the relation between imaging data and a non-destructive evaluation of electrical properties. Further, the methodology to capture datasets relating electrical properties of CFRP with increasing levels of surface abrasion can be replicated for different CFRP layups.

Specifications Table

**Table:** 

Subject	Electrical Power Systems and Carbon Fibre Reinforced Polymer (CFRP) Structures.
Specific subject area	Structural and Electrical Health Monitoring.
Data format	.csv (Direct electrical resistance of CFRP manually noted from impedance meter, frequency sweep dataset generated by Omicron Bode analyser software, surface roughness of CFRP generated by HIROX microscope software) .step and .igs (CAD file containing the 3D pot for the polishing machine) .tif (Microscopic images datasets generated from the HIROX microscope software).
Type of data	Numerical (Electrical resistance of unidirectional (UD) [0°] and woven CFRP samples at 0 Hz using an LCR meter (Direct current resistance (DCR) mode) and electrical impedance of the UD and woven CFRP samples with a range of frequencies, 1 Hz to 10 MHz; measured with an Omicron-Lab Vector Network Analyser - Bode 100 analyser. Measured, aggregated roughness of the surface of both the UD and woven CFRP samples. The arithmetic mean roughness (*R_a_*), maximum profile height (*R_z_*), and ten-point mean roughness height (*R_zjis_*), all in millimetres (mm) were measured using post processing with the HIROX Software Suite (version 1.5.6.0) using the slicer functionality to take a line scan across the centre of the 3D scan of potted CFRP samples). Images: (2D and 3D microscopic images captured using a HIROX 3D Digital Microscope RH-2000 digital microscope, and with HIROX Software Suite (version 1.5.6.0) using the semi-auto and standard quality settings, the in-build blended tiling method for 2D images and the auto 3D tiling setting for 3D images). CAD files (3D printed pot used to mount the UD and woven CFRP samples for polishing, electrical testing and microscopic analysis designed on SolidWorks) Drawing (2D drawing of Aluminium rig used to mount for measuring the electrical properties of the UD and woven CFRP samples).
Data collection	The electrical properties of CFRP, including impedance and phase angle, were measured using a frequency sweep (1 Hz – 10 MHz) with an Omicron Lab Vector Network Analyser - Bode 100 and DC (0 Hz) resistance measurements using the DCR mode of a BK Precision model 891, type 300 kHz Bench LCR meter. The microscopic images were captured using a HIROX RH-2000 digital microscope with 35x magnification. Microscopic images (2D and 3D) and surface roughness were captured using the bespoke software for the HIROX RH-2000 digital microscope (version 1.5.6.0). A BUEHLER AutoMet 300 Pro automatic grinder-polisher was utilised to abrade the CFRP samples UD samples were manufactured by the University of Strathclyde, and woven samples were provided by industry.
Data source location	Controlled abrasion of specimens, capture of microscopic images and roughness measurements of the CFRP samples were carried out at the Advanced Forming Research Centre, National Manufacturing Institute Scotland, University of Strathclyde, Glasgow, UK. Electrical measurements of the same CFRP samples were carried out in the Dynamic Power Systems Laboratory, Institute for Energy and Environment, Department of Electronic and Electrical Engineering, University of Strathclyde, Glasgow, UK.
Data accessibility	Repository name: Zenodo DOI: 10.5281/zenodo.20289363 Direct URL to data: https://zenodo.org/records/20289363
Related research article	M. Osama, C. E. Jones, and B. Stephen, “A predictive model for electrical fault uncertainty of carbon fibre reinforced polymer (CFRP),” in Proceedings of the Annual British Conference on Non-Destructive Testing, 61st Annual British Conference on Non-Destructive Testing, Sept. 2024, pp. 1–12. The British Institute of Non-Destructive Testing. DOI: 10.1784/ndt2024.4a1

## 1 Value of the Data

• This dataset enables estimation of the impact of CFRP surface condition (level of abrasion) on electrical fault response, which is necessary to inform the approach to fault management for resilient electrical power systems design, where electrical fault current may conduct through CFRP. This will subsequently enable design of electrical equipment where CFRP electrically couples to the electrical power system by forming part of the current return pathway for electrical fault current. In turn this allows for replacement of heavier metallic structures (e.g. equipment casings) with lighter CFRP structures, thus offering opportunity to increase power density of electrical power system equipment for weight critical applications, such as aerospace.
• The datasets capture the relationship between the duration of abrasion, change in surface properties (roughness, exposed carbon fibres) and the electrical resistance of CFRP for the two main, broad categories of pre-impregnated (pre-preg) CFRP used in aerospace structures: UD and woven.
• Knowledge of the range of resistance that CFRP may add to the conducting pathway taken by the electrical fault current is needed to support the resilient system design in safety-critical applications such as aerospace [1]. The datasets can be used to support development of modelling methods to predict the range of electrical resistance which the CFRP will add to the conducting pathway for electrical fault current to ground, as the surface of the CFRP is abraded. Abrasion will remove the outer layer of electrically insulating epoxy polymer matrix and exposing the electrically conductive carbon fibres, and hence electrical resistance will change [2].
• The dataset is of value to the following engineering communities:
  ○ Electrical power system engineers working in weight and safety critical applications who require CFRP electrical characterization datasets to inform design of electrical fault detection and protection systems where CFRP forms part of the conducting pathway for fault current.
  ○ Materials and manufacturing engineers to design and manufacture CFRP components for coupling to the electrical power system.
  ○ Metrology communities, including metrology standards and national metrology organisations, developing repeatable, reproducible methods to characterize changes in electrical properties of CFRP representative of in-situ degradation.
  ○ Aerospace original equipment manufacturers to support development and design of electrical power systems equipment and systems to minimise weight to reduce fuel burn and emissions.
  ○ Certification and industry standards bodies by informing new standards and regulations required to enable industry pull-through and commercial implementation of new, technologies where there is electrical coupling between the electrical power system and CFRP structures.

## 2 Background

The aviation industry contributes approximately 2.5% of global carbon dioxide emissions, which is expected to increase by 3.5% or more by 2050 [3]. Twin trends of increasing aircraft electrification and decarbonisation have emerged to overcome these challenges. The density of CFRP is ∼1.6 g/cm³ compared to ∼2.7 g/cm³ for aluminium. Hence, CFRP is used for over 50% of structures on state-of-the-art aircraft [4], offering a 20% weight reduction compared to aircraft with predominantly aluminium structures. This results in a 20% fuel burn reduction per aircraft [5]. Further opportunities exist to exploit the lightweight properties of CFRP for structures in electrical equipment such as casings for power electronic converters and electrical machines, where ∼10% weight is attributed to the aluminium casing [6]. Furthermore, cabling infrastructure to ensure CFRP is kept physically separate from cabling accounts for 30% of cable weight [7]. Closer physical integration of electrical equipment with CFRP via reduction in cable infrastructure and CFRP equipment casings, offers the opportunity to increase power density of the electrical power system for aerospace, but new failure modes where CFRP may conduct electrical fault currents must be understood, and appropriate fault mitigation strategies implemented.

The majority of electrical failures on aircraft are attributable to chaffed cables, caused by vibration [8]. Hence, the failure mode of concern is a cable chaffing against CFRP, damaging cable insulation and abrading the surface of CFRP, exposing carbon fibres. CFRP consists of electrically conducting carbon fibres held in an electrically isolating polymer matrix. As a consequence, the electrical properties are highly anisotropic. The electrical resistance added by CFRP to a fault path will vary depending on fibre lattice arrangement, distance travelled through the CFRP, and variation in ratio of polymer matrix and carbon fibre [1]. It will also be affected by the level of abrasion on the surface of the CFRP, which removes the outer layer of polymer resin to expose the carbon fibres [2]. An electrical protection system must be able to detect an electrical fault and respond in a timely manner before either the electrical power system or wider aircraft systems (including the structure) are damaged. Studies in [9] indicate that the electrical resistance of CFRP can be high enough that fault current magnitude is insufficient to trip electrical protection devices, but high enough for Joule heating of the CFRP to result in a temperature rise above the glass transition temperature of the polymer matrix, ultimately causing the CFRP to lose structural integrity.

Understanding and predicting the possible range of fault resistances provides valuable information to inform the approach to fault detection and location, which incorporates electrical power system architecture decisions to control fault response [1]. This paper presents a methodology for the capture of datasets which correlate the resistance of CFRP specimens with surface abrasion. In the initial experimental methodology, 320-grit paper was used to abrade the surface of CFRP by hand [9] . However, results in [2] indicated a high level of variation in the measured electrical resistance when CFRP was abraded by hand. It was unclear if this variation was due to systemic error introduced by using a manual abrasion method, or if it was due to physical differences between samples (e.g. position of fibres and polymer). Hence, this paper presents a new methodology which minimises systemic experimental error as far as possible, by use of automated methods to control the level of abrasion applied by controlled polishing of the CFRP surface, with controlled pressure and grinding speed. Further, an improved method to measure the resistance to reduce systemic measurement error as far as possible has also been developed. This paper presents these new methodologies and the associated datasets which they have been used to capture.

## 3 Data Description

**Fig. 1:**
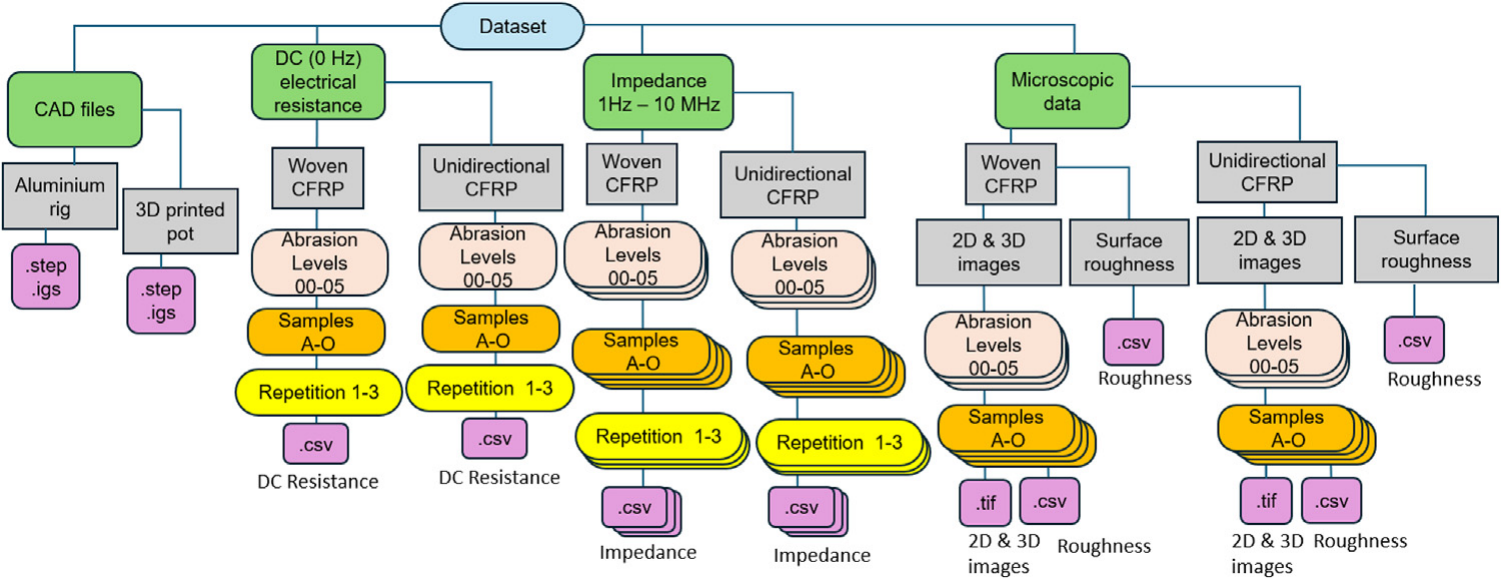
Graphical summary of the organisation of the dataset.

The dataset is divided into four sections, shown in Fig. 1: (i) CAD files, (ii) DC (0 Hz) electrical resistance, (iii) Impedance dataset over the frequency range 1 Hz – 10 MHz and (iv) surface roughness datasets. Fig. 1 shows the organization of the datasets and the file formats. Table 1 summarises the type and number of CFRP samples tested and the experimentally captured datasets.

**Table 1:** Summary of the experimental dataset structure, number of measurements made and units of measurement.

Dataset Description	Detail	Contents or Units
Sample information	CFRP Layups	UD [0°], Woven.
	Number of samples of each layup	15 (labelled A – O)
Abrasion information	Duration of application of abrasion for one abrasion level.	30 s
	Number of levels of abrasion	5
Electrical Datasets	Number of repetitions of electrical measurements per abrasion level for each sample.	3
	Units of electrical measurement for impedance and resistance.	Ohms
	Range of electrical frequency for impedance data	1 Hz – 10 MHz
	Frequency for DC measurements	0 Hz
	File format	.csv
Microscopic Datasets	2D Microscopic images	Graphical image of surface with stitched tiles.
	3D Microscopic images	Graphical image of surface with stitched tiles and z-direction variation in mm.
	File format of all microscopic images	.tif
	Aggregated roughness data	Ra (mm), Rz (mm), Rzjjs (mm)
	File format of roughness data	.csv

The CAD files are included in the dataset to enable replication of the experimental method. The CAD files contains design files for the aluminium test rig, which was used to measure the electrical resistance in a repeatable manner, and the 3D printed specimen pot which was used to hold the specimens during abrasion and electrical measurements. These files are provided in.step and .igs formats.

The experimental datasets are provided for two different layups of CFRP: woven and UD [0°]. For each layup, 15 samples were cut from the same panels of UD and woven CFRP. These samples were labelled A – O. The top surface of each sample was subjected to 5, 30 s periods of abrasion using the grinder-polishing machine, with each of these periods of abrasion referred to as a “level”. Data was collected for each level of abrasion, including prior to application of any abrasion. Hence for each sample there are datasets for 6 levels of abrasion (0 – 5, where 0 is no abrasion and 5 is 150 s total abrasion time).

Each electrical measurement was repeated 3 times for each sample for each level of abrasion. The electrical measurements were repeated to allow identification of systemic error. The DC (0 Hz) electrical resistance and electrical impedance over the frequency range 1 Hz to 10 MHz were both measured. In all measurement cases, the electrical measurements were measured between two, 5 mm x 5 mm electrode contact points on the surface of CFRP. The electrical measurement methods are described in detail in the Experimental Design, Materials and Methods section below.

Electrical measurements are provided as .csv file formats. For the DC Resistance measurements, there is one csv file of data correlates to one layup (woven or UD) and within the file all the DC resistance measurements for the 6 abrasion levels for all 15 samples (A – O) are listed. For the impedance measurements from 1 Hz – 10 MHz, for each layup and each level of abrasion and sample (A – O) there are 3 csv files of data, one for each of the three separate impedance measurements made. Hence there are 540 separate files for the impedance data.

Microscopic datasets are provided for all samples, woven and UD, for each level of abrasion. There is one folder with microscopic data for each sample, for each level of abrasion. Two sets of graphical images in .tif format are provided for each sample, for each of the 6 levels of abrasion: one is a 2D image of the surface, and two 3D images, one in original colour and one in pseudo colour. The 3D image shows variation in surface roughness in millimetres. Details of how these images were captured are described in the Experimental Design, Materials and Methods section below. For each sample a line profile scan was used to capture roughness data for a line running across the middle of the sample from one side to the other. The *R_a_* (arithmetic mean deviation of the measured surface profile, mm), *R_z_* (average maximum height of the sample profile, mm), and *Rzjis* (Japanese industrial standard (JIS B 0601)) for *R_z_*_which_ aggregates the 5 highest peaks and 5 lowest valleys over the sampled length). The roughness data is provided as a .csv file with data for each sample and abrasion level (same folder as the 2D and 3D images). The cross-sectional area, and dimensions over which the roughness was measured are also provided. In addition, for each layup, all roughness data for all samples and abrasion levels is also summarised in one .csv file. All units for roughness data are millimetres.

## 4 Experimental Design, Materials and Methods

### 4.1 Overview of the experimental process

The main objective of the experimental methodology presented in this paper is to progressively abrade the CFRP samples (UD and woven) and measure electrical impedance over the frequency range from DC (0 Hz) to 10 MHz and capture surface roughness using microscopic images as the level of abrasion increases. The datasets extracted using the presented methodology support correlation of changes in electrical resistance and the level of surface abrasion (duration in time and change in surface roughness levels). The 3D microscopic images visually indicate the level of surface roughness, and were used to provide quantitative values for the aggregated roughness of the surface of the CFRP samples.

Starting with non-abraded samples, progressive, stage-wise degradation was carried out by abrading the surface of CFRP using the grinder-polisher machine for 30 s per cycle, under constant parameters: applied pressure, grinding speed, contact force and feed rate. Both the grinding bed and the polishing holder rotated in the anticlockwise direction during polishing. Five cycles of polishing to abrade the surface were carried out, and after each stage, the DC (0 Hz) electrical resistance was measured using an LCR Meter set to the DC Resistance (0 Hz) function, a frequency sweep to measure electrical impedance from 1 Hz to 10 MHz was carried out using a Bode Analyser to measure electrical impedance. Microscopic 2D and 3D images were taken to observe visual changes in surface due to abrasion. The Microscopic 3D data also provided aggregated measurements of surface roughness (*R_a,_ R_z_, R_zjis_*) for a central line from one side to the other of a specimen (*x*-axis direction) crossing the centre of the sample.

## 5 Materials

### 5.1 CFRP samples

Two layups of CFRP were used. One layup was a UD [0°] layup with Toray T800 (polyacrylonitrile (PAN)) carbon fibre and SD400 epoxy polymer, and a volume fraction (*V_f_*) of 54%, with 22 plies (4 mm thick). The second layup was a woven layup with MTM44–1 epoxy resin and PAN carbon fibre, 8 plies (2 mm thick) and a *V_f_* of 58.5%. Samples were manufactured by laying up by hand and cured in an autoclave following the manufacturer’s cure cycle. 15 samples of each layup were cut. This number was chosen to be much higher than the minimum number of 5 test samples commonly recommended in industry test standards for CFRP to accommodate statistical variation between samples. The samples were all cut from one panel of material to eliminate variation caused by differences in environmental conditions during the manufacturing of different batches of CFRP panels. Samples were cut to be 25×25 mm using a Vitrex 180×16 mm diamond wheel blade saw. These sample dimensions were selected to fit into the available space for potted samples in the BUEHLER AutoMet 300 Pro automatic grinder-polisher machine. After cutting, the samples were wiped with acetone to remove any dust from the surface. Samples were stored in plastic containers packed with cotton wool for protection.

### 5.2 Methodology for potting CFRP samples

**Fig. 2:**
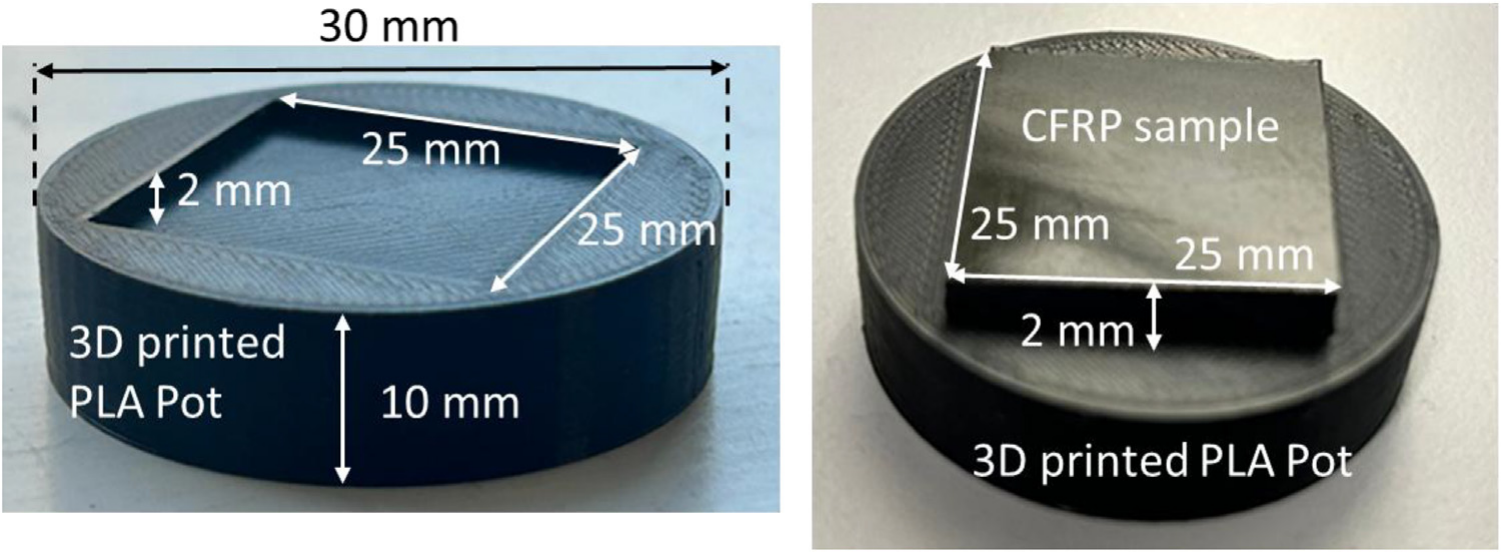
3D printed PLA pot for potting CFRP samples for the grinder-polisher machine, electrical and microscope measurements.

To address the issue of inconsistent application of pressure during manual abrasion [2] an experimental methodology was developed to abrade the top epoxy layer while maintaining consistent pressure throughout the machining process. To achieve this the abrasion process was automated using the BUEHLER, model AutoMet 300 Pro automatic grinder-polisher [10]. In order to mount the samples in the grinder-polisher machine, and to hold samples during electrical and microscopic testing, samples were potted. The size of the pot was determined by the size of the circular sample holders on the grinder-polisher. Samples were potted via manufacture of a 3D printed 30 mm diameter circular polylactic acid (PLA) thermoplastic pot (Fig. 2). Each PLA pot had a slot measuring 25 mm by 25 mm and a depth which was half that of the thickness of the CFRP samples (2 mm for the UD samples,1 mm for the woven samples) to hold the CFRP securely. The surface of the CFRP sample sits above the surface of the pot (2 mm for UD samples, 1 mm for woven samples) to ensure the surface of the sample was polished, rather than the pot surface.

## 6 Surface Abrasion Methodology

### 6.1 Polishing machine setup

**Fig. 3:**
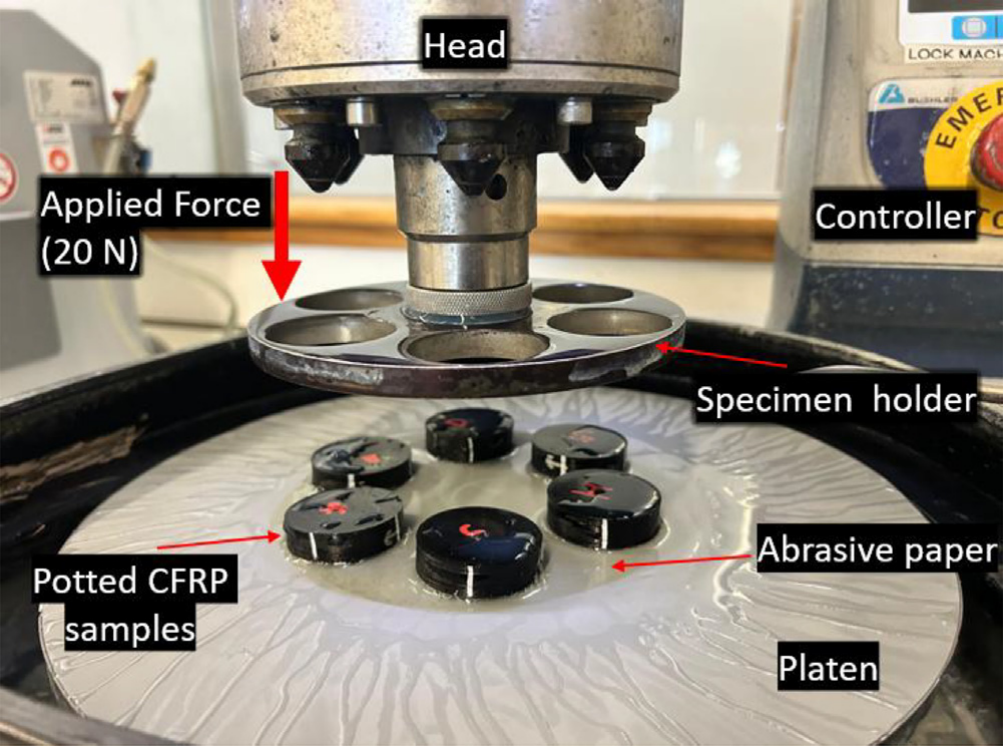
Potted CFRP samples on the grinder-polisher machine, with the CFRP surface on the abrasive paper. During the abrasion process, the potted samples are held by the specimen holder.

Up to 6 CFRP samples could be abraded at one time using the BUEHLER grinder-polisher. The potted samples were placed in the sample holder with the CFRP samples facing downwards, such that the CFRP surface was held against the abrasive paper mounted on the platen, as shown in Fig. 3: Potted CFRP samples on the grinder-polisher machine, with the CFRP surface on the abrasive paper. During the abrasion process, the potted samples are held by the specimen holder. . Abrasive paper P2500 of. 8.4 µm thickness, standard diameter 304.8 mm was used. The process for selecting the grade of abrasive paper is described in Section 3.2. Before attaching the abrasive paper, a BUEHLER releasing agent 20–8186–004 [11] was applied to the surface of the grinding platen using the applicator that the release agent comes with. The release agent was spread over the surface of the platen using a releasing agent brush. The release agent allowed the paper to be easily removed after the experiment and prevents any adhesive residue from being left on the platen. A constant force of 20 N was applied with a head speed of 60 RPM and a platen speed of 250 RPM for 30 s during each cycle of abrasion. Water was applied during polishing to prevent CFRP dust particles entering into the air in the laboratory and to minimise the temperature rise of the sample during abrasion.

### 6.2 Methodology to select the of Grade of abrasion paper

To select the grit level of abrasion (sand) paper to use to abrade the samples, two grades of silicon carbide abrasion paper were selected for comparison: P2500 and P1200 and levels of abrasion. Five samples were polished with P1200 silicon carbide abrasion paper, and five were polished using P2500 silicon carbide abrasion paper. For each set of samples, 3 rounds of abrasion were applied. After each abrasion cycle, all samples were dried using an air-dryer in the laboratory. Before the first abrasion cycle (referred to as “Level 1” in the dataset) and after each subsequent abrasion cycle, the electrical resistance and impedance of the samples were measured using the method described in Section 5. As a result of these tests, P2500 silicon carbide abrading paper was selected, as it provided a greater granularity in the change in resistance with respect to the number of abrasion cycles.

## 7 Electrical Measurement Methodology

The DC (0 Hz) electrical resistance was measured using the BK Precision model 891, type 300 kHz Bench LCR meter [12], operated in DC resistance (DCR) mode, corresponding to a true 0 Hz measurement condition. For the DC resistance measurements, the 4-point measurement method was applied by using Kelvin probes with the LCR meter. Electrical impedance measurement over the frequency range 1 Hz – 10 MHz was measured using the Omicron Lab Vector Network Analyser - Bode 100 with Bode analyser suite version 3.25.2267.0002 software, build data 2023–08–29 11:54:06z [13]. For the Bode analyser the 4-point measurement method was applied by operating the Bode analyser in the shunt-thru configuration. Instrument calibration for both the LCR meter and the Bode Analyser was performed for short-circuit, open-circuit and load calibration using a known resistor, 25 Ω.

**Fig. 4:**
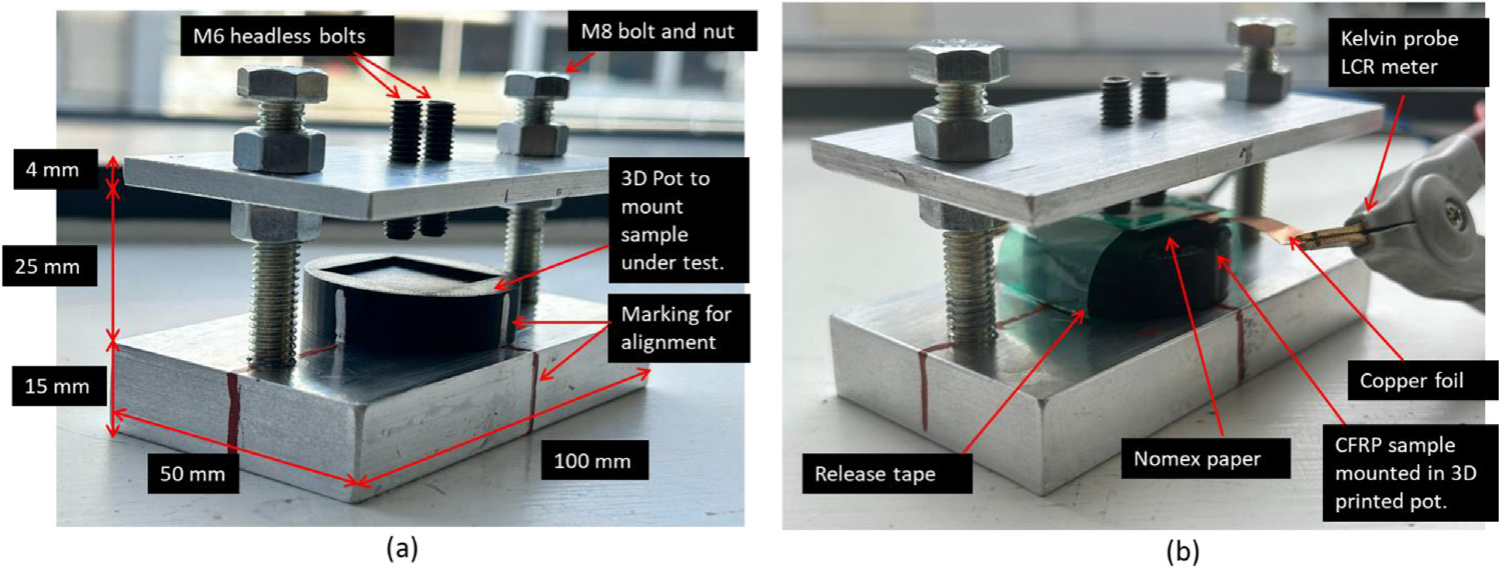
Aluminum rig for electrical measurement: (a) indicates rig dimensions and positioning of 3D pot to hold the sample during test; (b) shows set up with a sample under test. Set up for electrodes on the surface of the CFRP is detailed in Fig. 5.

The aluminium test rig in Fig. 4 was used to hold the samples during electrical testing. To ensure that electrical impedance was measured between the same points on the surface of the pot into the rig, visual markers were positioned on the 3D pot and the test rig (indicated in Fig. 4).

**Fig. 5:**
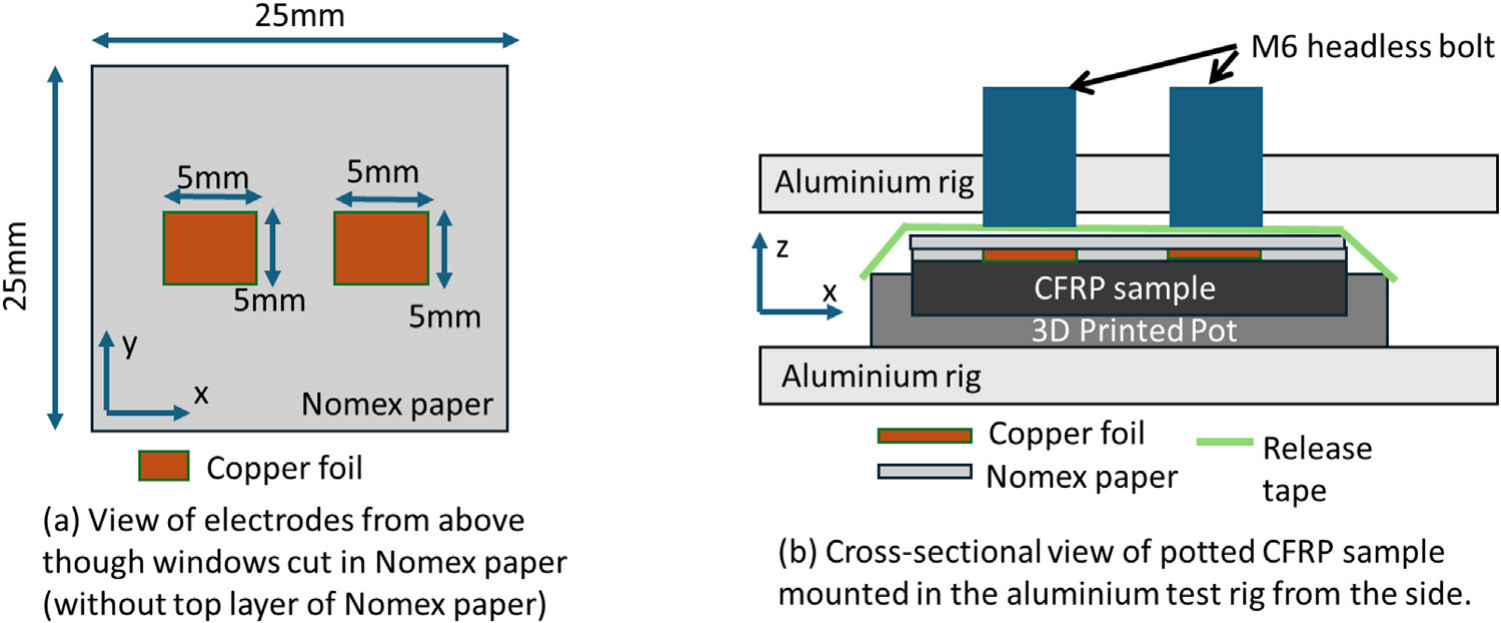
(a) diagram of electrodes created by cutting a 5 × 5 mm window in Nomex paper, with copper foil placed on the upper side, as indicated in (b) which is the cross-sectional view of the electrical measurement set-up of the potted sample in the rig.

Fig. 5 shows the set up for connecting the copper foil electrodes to the surface of the CFRP. To measure the electrical resistance between two, 5 × 5 mm areas on the abraded surface of the CFRP samples, a piece of Nomex paper [14] had two 5 × 5 mm windows cut in it (Fig. 5). Two strips (one for each electrode) of 0.1 mm thick annealed copper foil were placed onto the Nomex paper. The windows in the Nomex paper ensured that electrical contact between the CFRP and copper foil was only made over the 5 × 5 mm windows. A second piece of Nomex paper was placed over the top of the copper foil electrodes (no windows were cut in this piece of Nomex paper). Release tape was placed over this second layer of Nomex paper to hold the paper and copper foil in place (Fig. 5). The copper foil electrodes were cleaned before each measurement to remove any layer of oxidation with wire wool. The second layer of Nomex paper provided electrical insulation between the copper foil electrodes and the M6 headless bolts which were used to apply a controlled force to the two, 5 × 5 mm measurement points. The application of the same force to electrodes for each measurement was achieved by ensuring that the M6 bolts were turned the same number of times when setting up to take each electrical measurement. The Kelvin probes for the DC resistance measurements and the crocodile clips for the connection to the Bode analyser for the impedance measurements were clipped to the copper foil at the side of the aluminium test rig (Fig. 4).

**Fig. 6:**
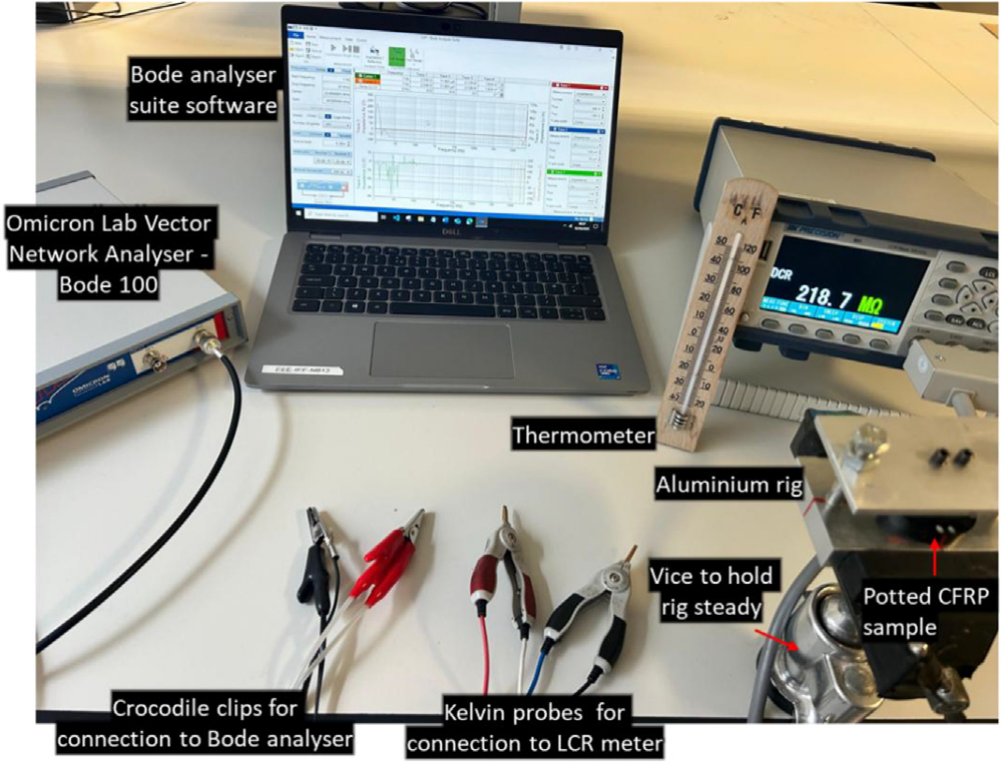
Overview of bench-top set up for electrical testing of samples for both DC (0 Hz) using the LCR meter and impedance using the Omicron Lab Vector Network Analyser- Bode 100.

Fig. 6 shows the experimental set up on the laboratory bench during electrical testing. A benchtop clamp was used to hold the aluminium test rig securely during testing, as indicated in Fig. 6. DC resistance values using the LCR meter were recorded by hand. The impedance measurements from the Bode analyser were recorded using the laptop with Bode analyser suite software.

The electrical measurement process was repeated 3 times for each sample for each level of abrasion. Ambient laboratory temperature was measured using a thermometer to ensure that any variation in laboratory temperature was known. Ambient laboratory temperature measured, during testing and remained at 22 ± 1 °C during the course of the measurements.

## 8 Surface Roughness and Microscopic Analysis

**Fig. 7:**
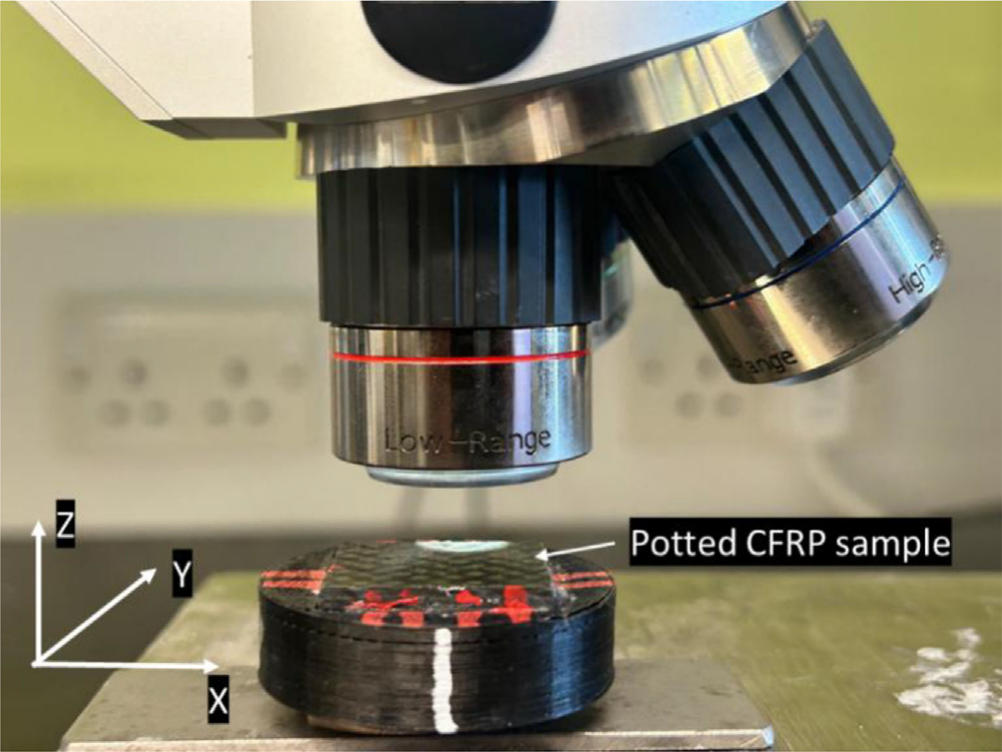
HIROX microscope set up to scan a potted CFRP sample.

The 2D and 3D Microscopic images and roughness data were captured using a HIROX RH-2000 digital microscope [15], shown in Fig. 7, with the HIROX Software Suite (version 1.5.6.0). The measurements utilised a low-range ×35 lens MXB-2500REZ with a field of view of 31,931 µm and a resolution of 4 µm. The lighting intensity level of the HIROX microscope was set to level 100, with standard gamma adjustment and auto shutter speed.

To capture the 2D images blending tiling method of the microscope software was used. “Standard” settings for pixel size were used (1440×900), with the standard auto tiling setting. The final size of each tiled image was 7440×6855 pixels. To create a 3D image the Auto 3D Tiling setting was used. Pixel size was again set to be “Standard”. The stitching setting was set to “Standard” and the blending tiling method selected. To ensure the full CFRP sample was scanned for both the 2D and 3D images, the scanned area was set to extend beyond the edge of the CFRP sample to the 3D printed pot that the samples were mounted in Post processing was used to generate the 3D images of the 3D tiled images, and to measure roughness using the HIROX Software Suite. The images were generated for the dataset both in original colour and pseudo colour (using the settings in the software suite).

Roughness was measured from the generated 3D images. Using the HIROX Software Suite, a slider was positioned across the centre of the sample, running parallel to the y-axis. The surface area of the slice used to measure the roughness is not held constant; this was set automatically by the HIROX Software Suite. The aggregated roughness measurements for arithmetic mean roughness (*R_a_*), maximum profile height (*R_z_*) and ten-point mean roughness height (*R_zjis_*) were calculated using the HIROX software suite. A limitation of the roughness measurements is that the pot in which the CFRP samples are mounted and height difference between the CFRP sample surface and the pot surface (which is a step of 2 mm for the UD samples, 1 mm for the woven samples) is included in the aggregated data. Further analysis is needed to assess whether this bias is constant for all roughness measurements, and whether therefore it can be accounted for in the roughness assessment and comparison.

## 9 Management of Uncertainty and Variability of the Dataset

Management of uncertainty and variation in the experimental dataset heavily influenced the experimental design and methodology for the capture of the datasets. Two types of uncertainty are defined in the literature: Type A (aleatoric) which is driven by randomness, and Type B (epistemic) which is driven by lack of knowledge [16]. The experimental method was designed to minimise aleatoric uncertainty as far as possible: samples were all cut from the same panel (to minimise manufacturing variation); an automated methodology was applied to progressively abrade the surface of the CFRP; the electrical measurement rig ensured that electrical properties were always measured between the same points on the surface of the CFRP with the same pressure applied; the 4-point measurement method eliminated cable impedance; electrical properties were measured 3 times for both DC resistance and impedance measurements.

**Fig. 8:**
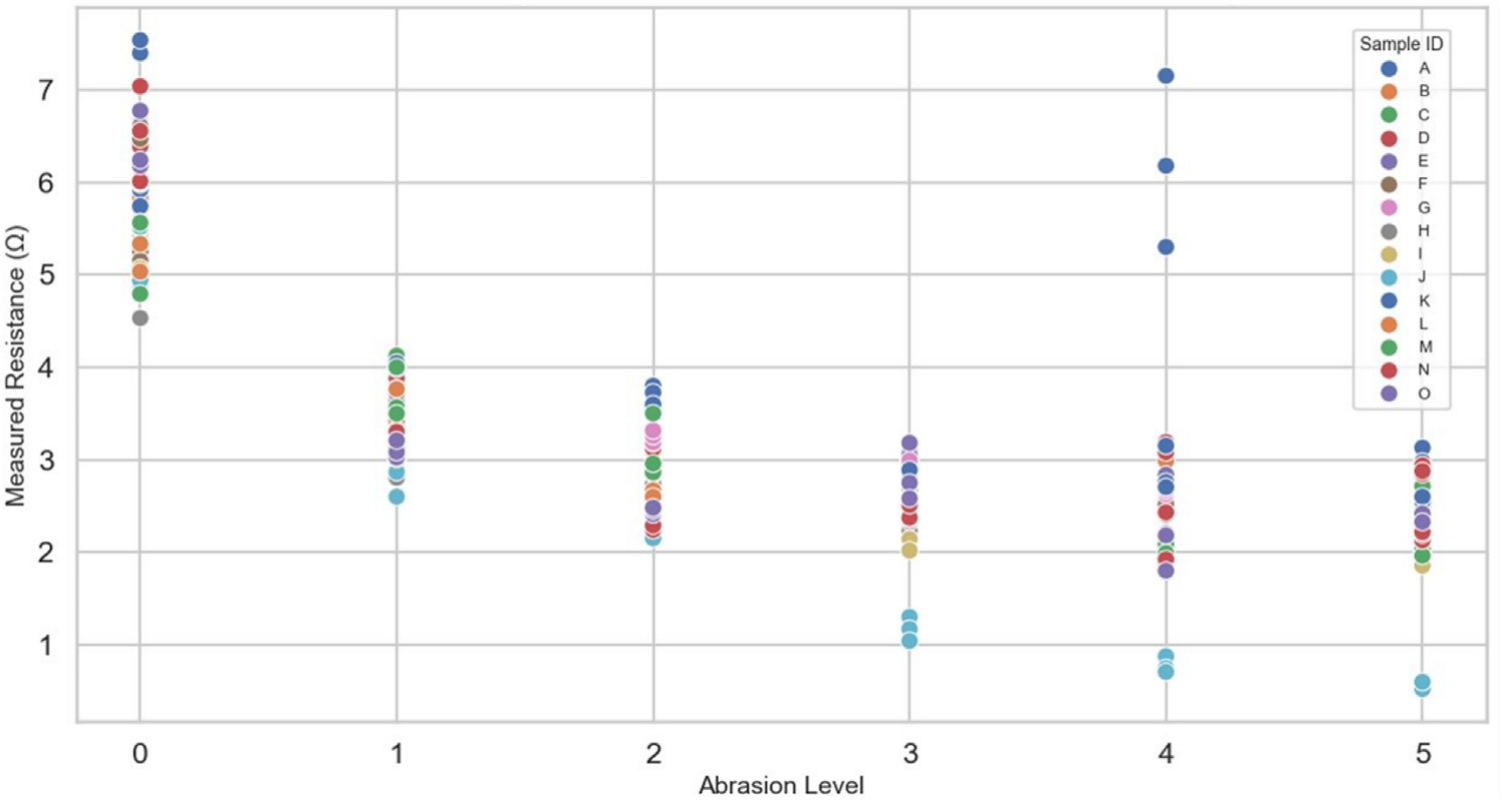
Variation of DC resistance of UD samples with level of surface abrasion.

**Fig. 9:**
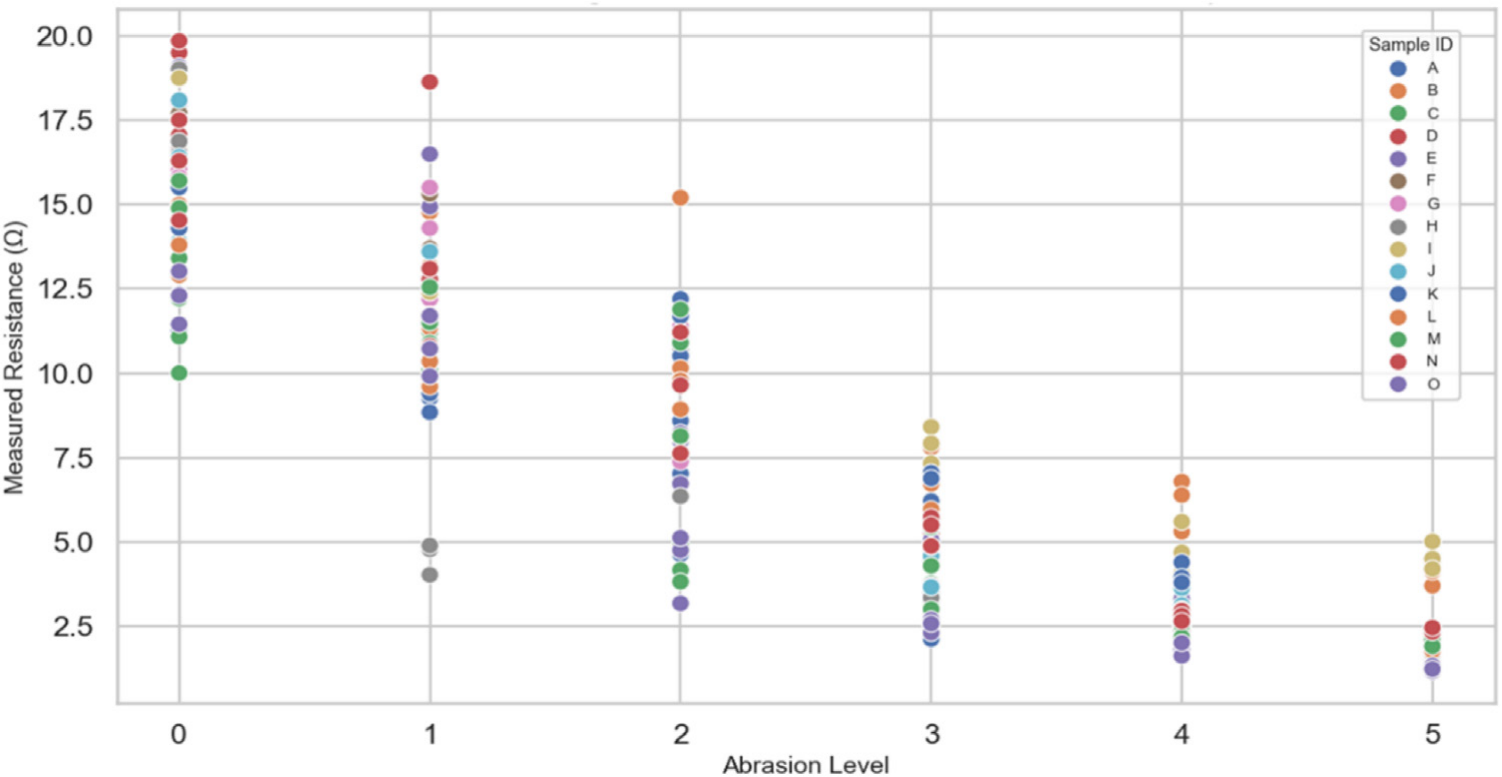
Variation of DC resistance of woven samples with level of surface abrasion.

To demonstrate the impact of measures taken to minimise aleatoric uncertainty, Python-based code was used to visualise the relationship between the measured electrical DC resistance and abrasion level of both layups, UD (Fig. 8) and woven (Fig. 9) CFRP. All the sample IDs are labelled; the same colour points indicate the repetition of the electrical resistance measurement being taken three times at a particular abrasion level for a particular sample. Of particular interest in Fig. 8 are that the measured resistances for Sample J for 3 levels of abrasion have consistently lower resistance than the other samples. Sample A has a much higher resistance at abrasion Level 4 compared to other samples. Cross-checking with the impedance datasets for both sample J and sample A at abrasion level 4, the real component of the impedance at 1 Hz in both cases is within 0.1 Ω of the DC resistances measured. These values are an example of epistemic uncertainty in the dataset.

**Fig. 10:**
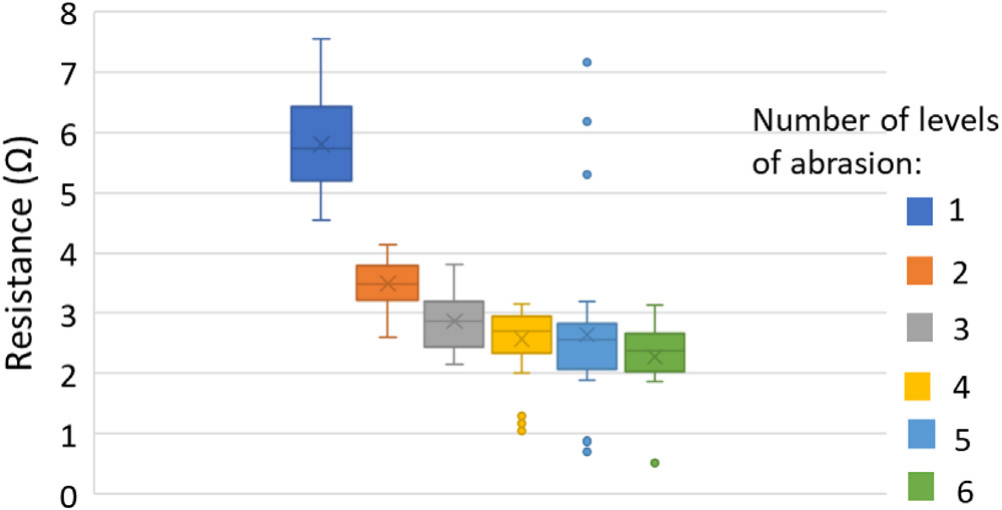
Box and whisker plot of measured DC electrical resistance for all UD samples for each level of abrasion.

**Fig. 11:**
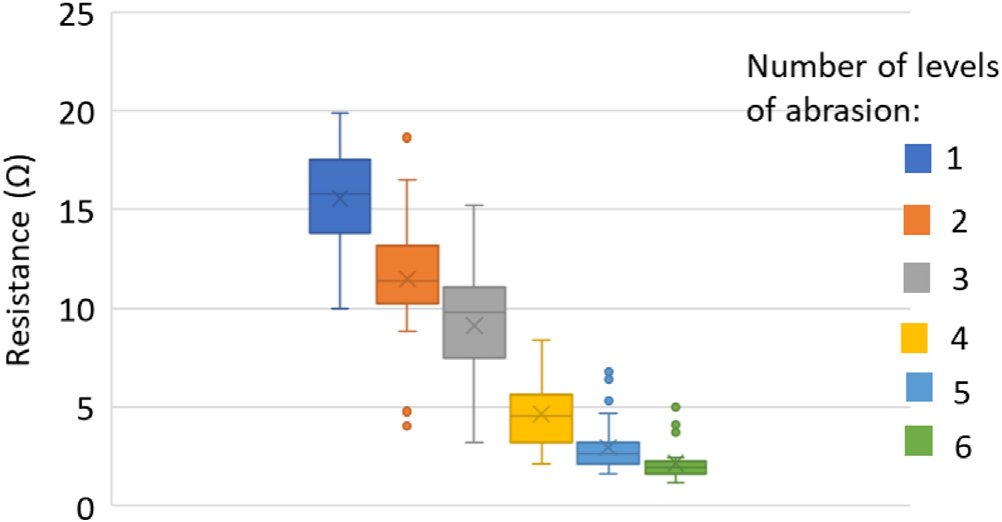
Box and whisker plot of measured DC electrical resistance for all woven samples for each level of abrasion.

The variability of the measured DC resistances for each level of abrasion for both the UD and woven CFRP samples was analysed using a box and whisker plot, plotted using Excel. These are shown in Fig. 10 for UD, and Fig. 11 for woven. In Fig. 10 the lower resistances for Sample J can be identified at levels of abrasion 3–5. The high resistance outliers at Level 5 are attributed to Sample A. A general trend is that the UD samples have a lower level of variability compared to woven. Outliers with higher resistance at Levels 4 and 5 for woven are attributable to samples L and I. There are only two outliers at Levels 1 and 2 for woven which are single measurements for one specific sample. This demonstrates that the DC resistance data capture methodology reduces random uncertainty, and that the results have good repeatability for each single sample, and good reproducibility across different samples. This indicates that the higher variation in the woven results is driven by variations between physical attributes of the samples, rather than uncertainty introduced by the data capture method.

The microscopic images and roughness data provide capability to investigate the correlations between changes in electrical resistance and physical changes to the surface of the CFRP specimens due to abrasion. This includes investigation of the cause of the outliers identified from DC resistance data analysis further. For example, with reference to Fig. 8, UD Sample J for abrasion levels 3 and above, and UD Sample A at abrasion level 4.

**Fig. 12:**
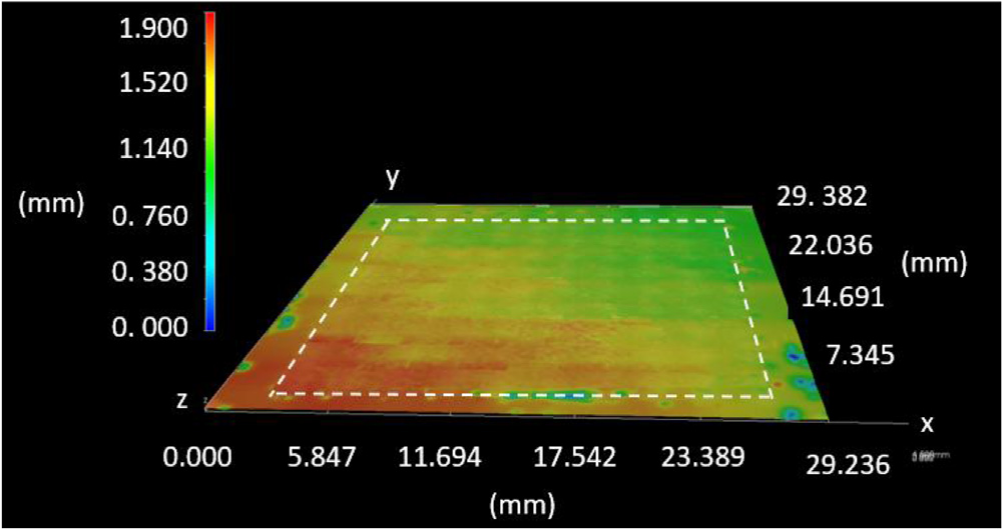
3D microscopic images of surface degradation of UD sample “J” after 4 cycles of abrasion.

**Fig. 13:**
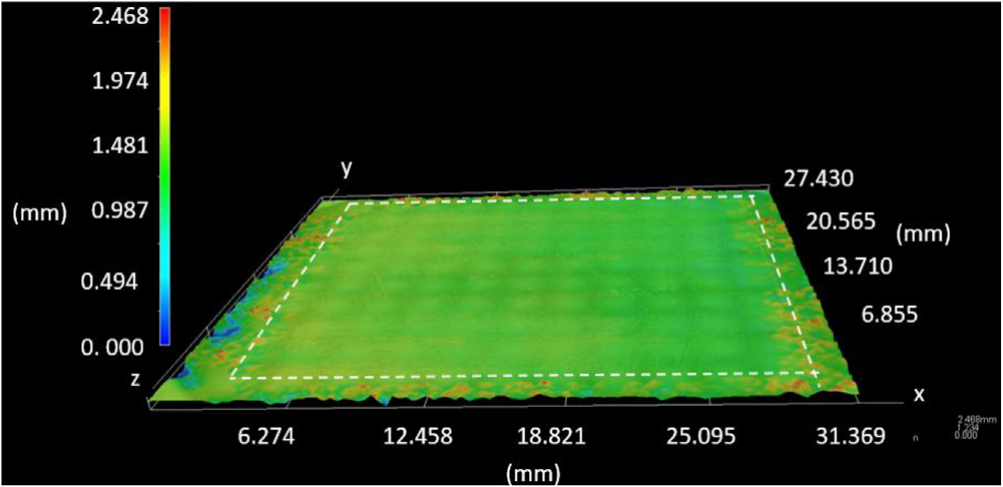
3D microscopic images of surface degradation of UD sample “A” after 4 cycles of abrasion.

**Fig. 14:**
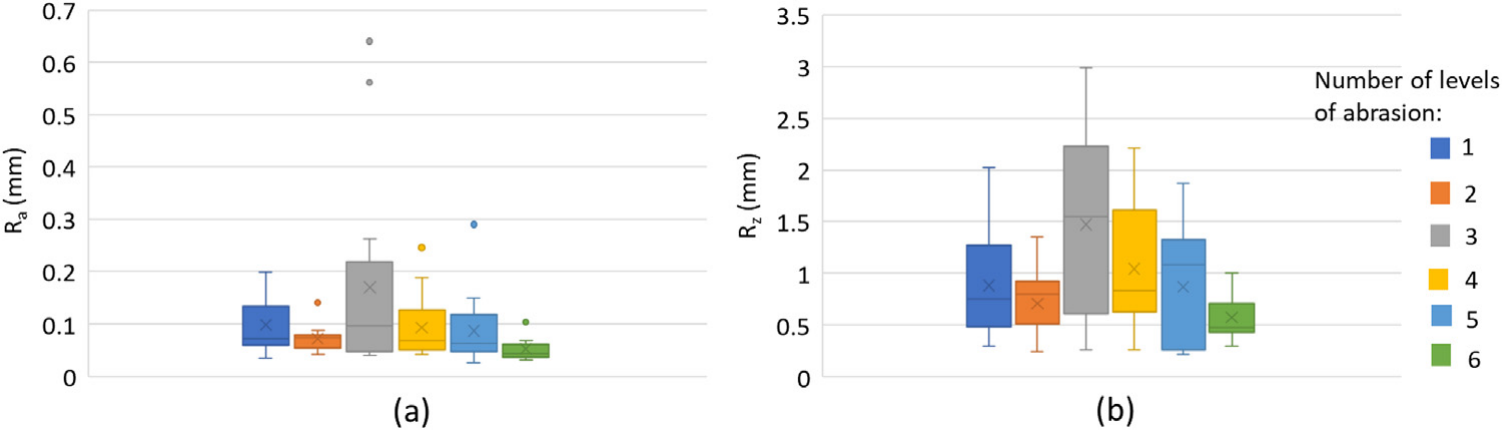
Box and whisker plot of measured roughness for all UD samples: (a) R_a_ and (b) R_z_.

The 3D scanned image of the surface of UD sample J after 4 rounds of abrasion is shown in Fig. 12. The 3D scanned image for sample A after 4 cycles of abrasion is shown in Fig. 13. The dashed lines (added to the figures for this paper) in both sets of results indicate the edge of the scanned CFRP samples, with the CFRP within the area enclosed by the dashed lines. By inspection of these 3D images, a higher level of variability in surface roughness is indicated for sample J compared to sample A. This observation does not by itself explain the reason why the measured resistance of sample J is ∼1 Ω (>50%) lower than that of sample A. However, it does indicate that application of image processing methods to the microscopic images may enable quantifiable reasoning for an outlying resistance value compared to other CFRP samples, for example to quantify the ratio of exposed, electrically carbon fibres on the CFRP surface to the amount of electrically insulating polymer matrix Fig. 14.

**Fig. 15:**
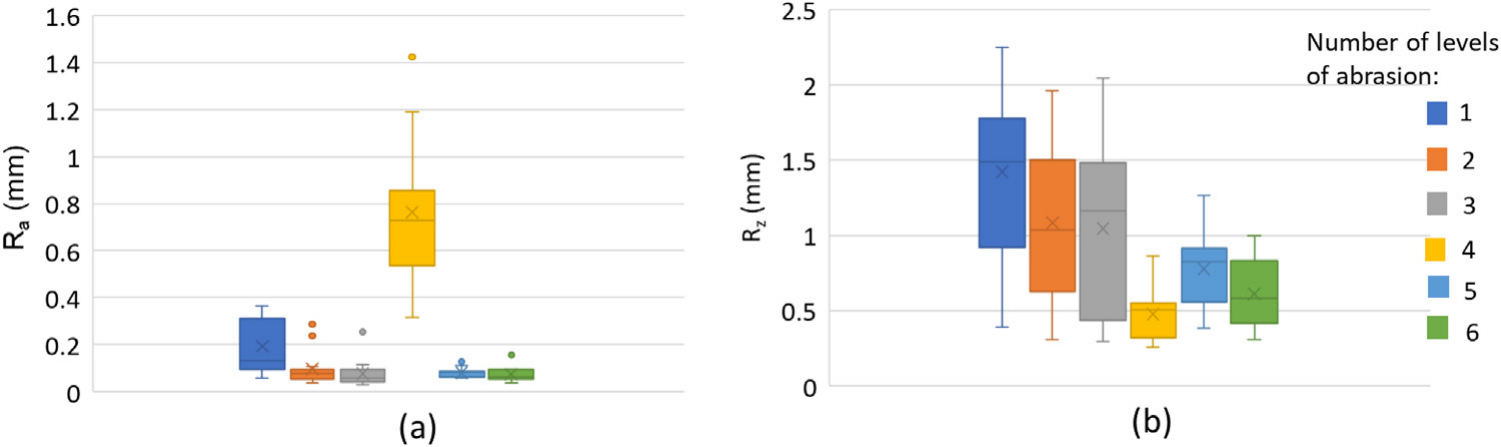
Box and whisker plot of measured roughness for all woven samples: (a) R_a_ and (b) R_z_.

The variation in roughness, R_a_ and R_z_, is shown in for all the UD samples and Fig. 15 for all woven samples. For both layups the data indicates high variation in levels of variability in roughness. For UD there is correlation between level of variation, and trends in mean values between R_a_ and R_z_. there is much lower variation (with exception of Level 4) in Ra values for woven compared to UD. However there is no correlation between woven values for Ra and Rz. As described in Section 6, the roughness values vary in surface area, and includes the surface of the pot that the sample is potted in This may explain the high values for R_z_ compared to R_a_, and adding a constant bias to the data.

## Limitations

The datasets are limited first by the fact that only two layups have been tested. Therefore sensitivity of properties captured by the dataset to different carbon fibre orientations, type of carbon fibre (pitch or PAN), type of resin, additives and method of manufacture is not captured by the dataset. However, the experimental methodology described provides an opportunity to derive datasets for other CFRP layups.

A second limitation is that the sample size was limited to 25×25 mm due to the polishing machine constraint. Further work is needed to investigate methods to deliver datasets for larger sized samples of CFRP, which are more representative of the size of CFRP components, and establish whether results are scalable to bigger components.

Abrasion of the surface of a CFRP sample changes the electrical characteristics of the CFRP due to removal of electrically insulating polymer matrix from the surface of the CFRP, exposing the electrically conductive carbon fibres. A limitation of the dataset is that further processing of the data is needed to correlate the observed changes in electrical resistance to physical changes of the abraded surface (2D, 3D microscopic images and roughness datasets) of the CFRP in a quantifiable manner.

The 3D scanned images in the dataset include the surface area of the CFRP and the surrounding 3D pot in which the CFRP samples are mounted. The subsequent post processing of these images to measure surface roughness has included the measurements of the surface of the pot in the aggregated results. Further investigation of this dataset is needed to establish whether the inclusion of the pot surface adds a constant bias to the measured roughness which can be accounted for.

A further limitation is that the datasets provide a single value for the resistance of the conducting pathway through the CFRP. The aim of this dataset was to characterise overall resistance change with abrasion, as it is total fault resistance which influences electrical power system fault response. However, the resistance of the CFRP will vary along the conducting pathway [1,17]. Electrical resistance is higher if current flows through a smaller cross-sectional area. Carbon fibres in CFRP will touch within plies and between plies, forming electrical connections. If the CFRP is considered at a macroscale and considered as a homogeneous material, then electrical current conducts though a cross-sectional area determined by electrode size at the points where it enters and exits the CFRP. As the current flows through the CFRP, the cross-sectional conducting area increases due to the inter and intra ply connections between carbon fibres. Therefore electrical resistance at input and output is higher at electrodes compared to the conducting pathway between electrodes due to the smaller cross-sectional area of conduction. This input and output resistance are also increased due to contact resistance between the copper electrode and the CFRP. A limitation of the dataset is that these variations in resistance along the conducting pathway are not captured. Further work is needed to develop metrology methods to measure this variation in resistance. Extension of the dataset in this way would subsequently inform scalability of results, by quantifying the influence of input and output resistance on total resistance. Ultimately if input and output resistance dominate, then total resistance is not sensitive to panel size.

A second limitation of missing data for variation of resistance along the conducing pathway closely links to a further limitation which is that the dataset does not include electrical-thermal response. Knowledge of how abrasion, magnitude of fault current and Joule Heating is important to fully understand the full fault response, and the speed with which electrical protection must respond to a fault to prevent damage to the CFRP due to high levels of Joule Heating, caused by resistive losses during electrical conduction which are dissipated as heat. The level of Joule Heating will be higher at points on the conducting pathway where localised electrical resistance is higher. Without knowledge of the variation of electrical resistance, it is not possible to estimate expected power dissipation, and correlate this to temperature change, at different points on the conducting pathway. High levels of localised Joule Heating can lead to the glass transition temperature of the polymer matrix being breached and subsequent thermal degradation of the CFRP component.

In real electrical faults through CFRP, electrical current may enter through an abraded area of CFRP but exit via a point where the CFRP is electrically bonded through a fastener to electrical ground, or current return, such as described in [12]. This will affect the pathway taken by electrical current through the CFRP, and hence impact on both total resistance, and localised resistance at the output terminal in particular. Future work is needed to expand the dataset to incorporate electrical bonding, and combine this with data on variation of electrical resistance along the conducting pathway, and Joule Heating. This would inform design and manufacture of methods for electrical bonding to CFRP, and inform limits for electrical current magnitude, duration and electrical resistance of these methods.

Furthermore, all experiments were conducted in an idealised laboratory environment, at room temperature (22 ± 1 °C) and sea-level air pressure with clean, samples free of any surface contamination (e.g. oil). Influence of variation of air temperature, air pressure, moisture level and surface contamination on electrical resistance were not included. Further investigation of experimental methods for replication of fault manifestation in the laboratory environment to capture sensitivity of electrical resistance and level of abrasion to environmental conditions is needed. For temperature, moisture and pressure, an environmental test chamber would enable this. For contamination, consideration of types of contaminant must be considered, and methods to apply these which replicate in-situ conditions in the laboratory implemented. For the dataset presented, a constant force was applied to the electrode surface. This follows the experimental methodology in [12]. However, for an in-situ failure, constant force may not be applied to the cable against the CFRP. To overcome this limitation, first a future dataset must be captured to capture variation of resistance with applied force for different levels of abrasion. A further consideration is the method by which the abrasion has been applied for the dataset. The polishing machine was chosen to minimise variation of level of abrasion applied to each sample. However, in practice abrasion will be due to a loose wire chaffing against a CFRP component due to vibration [8]. Further work to develop a repeatable, reproducible method to replicate this in the laboratory is needed.

## Ethics Statement

This research did not involve human subjects, animal experiments, or data collected from social media platforms. Authors have read and followed the ethical requirements for publication in Data in Brief*.*

## CRediT authorship contribution statement

**Muhammad Osama:** Conceptualization, Data curation, Methodology, Software, Investigation, Resources. **Catherine E Jones:** Supervision, Conceptualization, Data curation, Project administration. **Bruce Stephen:** Supervision, Conceptualization, Project administration.

## Data Availability

Zenodo.Electrical Properties and Surface Roughness of Carbon Fibre Reinforced Polymer Under Progressive Surface Abrasion (Original data)
